# Postoperative Pain Medication Utilization in Pediatric Patients Undergoing Sports Orthopaedic Surgery: Characterizing Patient Usage Patterns and Opioid Retention

**DOI:** 10.5435/JAAOSGlobal-D-22-00206

**Published:** 2022-10-24

**Authors:** Allan K. Metz, Kelly M. Tomasevich, Devin L. Froerer, Reece M. Rosenthal, Joseph Featherall, Stephen K. Aoki

**Affiliations:** From the Department of Orthopaedics, University of Utah, Salt Lake City, UT (Metz, Dr. Tomasevich, Rosenthal, Dr. Featherall, and Dr. Aoki), and the School of Medicine, University of Utah, Salt Lake City, UT (Froerer).

## Abstract

Opioid overprescribing is a problem in orthopaedic surgery, with many patients having leftover opioid medications after surgery. The purpose of our study was to capture utilization patterns of opioids in pediatric patients undergoing orthopaedic sports medicine surgery, in addition to evaluating patient practices surrounding unutilized opioid medication. Our hypothesis was that there would be low utilization of opioids in this patient population and would in turn contribute to notable overprescribing of opioids and opioid retention in this population.

**Methods:**

Pediatric patients undergoing orthopaedic surgery for knee and hip pathology were prospectively enrolled. A survey was administered 14 days postoperatively, with questions centered on the patient-reported number of opioids prescribed, number of opioids used, number of days opioids were used, and incidences of leftover opioid medication and disposal of leftover medication. The magnitude of opioid overprescribing was calculated using the reported prescribed and reported used number of opioid pills. Linear regression was used to examine associations between opioids and NSAIDs prescribed.

**Results:**

One hundred fourteen patients reported a mean prescription of 12.0 ± 5.0 pills, with utilization of 4.4 ± 6.1 pills over 2.7 ± 5.1 days. Patients were prescribed 2.73 times the number of opioid pills required on average. One hundred patients (87.7%) reported having unused opioid medication after their surgery, with 71 (71.0%) reporting opioid retention. Regression results showed an association with opioids used and prescribed opioid amount (β = 0.582, R = 0.471, *P* < 0.001).

**Discussion:**

Overall, our study results help characterize the utilization patterns of opioid medications in the postsurgical pediatric sports orthopaedic population and suggest that orthopaedic surgeons may be able to provide smaller quantities of opioid pills for analgesia than is typically prescribed, which in turn may help reduce the amount of prescription opioid medications present in the community.

**Level of Evidence:**

Level IV

The opioid epidemic in the United States (US) continues to worsen despite concerted efforts to curtail it, with the Centers for Disease Control and Prevention estimating that prescription opioid overdose-related deaths have increased approximately fourfold since 1999.^1,2^ Although there has been a recent cultural change and a concerted effort to curtail opioid abuse, a notable driver of the opioid epidemic remains the misuse of prescription opioid medications, which are often initiated in the perioperative setting.^3^ Misuse of these medications has been estimated to generate notable costs to the US healthcare system, with some estimates placing the figure as high as 78.5 billion US dollars annually.^4^ Recent estimates report that up to 4% of the US population misuses prescription opioids, with the overwhelming majority beginning this misuse because of chronic pain often stemming from surgical procedures.^5,6^ Opioid methods of analgesia are used more frequently in orthopaedic surgical practice than in other surgical specialties, with opioid analgesia frequently initiated in the perioperative setting.^3,7,8,9,10,11^ Although opioids are an effective modality of pain management in orthopaedics and may provide benefit to patients by reducing pain and increasing their ability to participate in postoperative therapy, the risk of abuse and addiction cannot be ignored.

A recent increase has been observed in opioid usage research in an attempt to better understand this epidemic, with an even more recent focus on opioid medication usage in the pediatric population. Investigations into the opioid prescribing patterns of surgeons, especially in orthopaedic surgery, has shed light on potential avenues to address the US opioid epidemic, the most concerning of which being overprescription of these medications, especially within the pediatric population. Recent research has demonstrated a high potential for community opioid retention in this group, potentially stemming from better recovery and pain control in children and adolescents when compared with adults undergoing similar surgeries.^12-14^

The purpose of our study was to capture utilization patterns of opioids in pediatric patients undergoing orthopaedic sports medicine surgery, in addition to evaluating patient practices surrounding unutilized opioid medication. We hypothesized that there would be low utilization of opioid pain medication in this patient population and that would in turn lead to notable overprescribing of opioids in this population and a notable percentage of opioid retention in the community within our study cohort.

## Methods

### Data Collection, Cohort Identification, and Survey

After obtaining IRB approval (#137113), pediatric patients undergoing surgery by the senior author (S.K.A.) were prospectively identified from February 22, 2021, to November 1, 2021. Patients underwent either arthroscopic knee procedures (medial patellofemoral ligament reconstruction, cruciate and/or collateral ligament repair, meniscus débridement and/or repair, treatment of osteochondritis dissecans, hardware removal, etc) or arthroscopic hip procedures (osteochondroplasty for femoroacetabular impingement syndrome, labral repair for labral tears, etc) to address their presenting pathology. Demographic information such as patient age at surgery and sex were collected from the institution's electronic medical record. Inclusion criteria for this study were as follows: (1) patient age 7 to 17 years at the time of surgery, (2) knee or hip surgery by the senior author, and (3) consent for survey administration at 14 days postoperatively.

Patients and their families were consented for participation in a survey to evaluate their postoperative pain medication usage in the preoperative setting. Questions, which are listed in full in the Supplemental Appendix (http://links.lww.com/JG9/A236), included asking patients to report the number of opioid pills they used, the number of days they used opioid pills, whether they had opioid pills remaining after surgery, and whether they disposed of excess opioid pills. The number of opioid pills prescribed was retrieved from the institution's electronic medical record, along with any requests for additional opioid medication by refills. The survey was administered 14 days after surgery, and survey data were collected and managed using Research Electronic Data Capture electronic tools (Vanderbilt University) by e-mail. Because our study focused on the utilization of opioid medications in pediatric patients, the survey was sent to the parent(s) and/or guardian(s) of the patient with encouragement for the survey to be completed together to ensure accurate reporting of medication usage. Survey reminders were sent through e-mail for 1 month, after which time, families were contacted through phone for a maximum of three calls to promote survey completion.

### Postoperative Analgesia Protocol

All patients included in this study received at least the same three drug classes as part of their postoperative medication regimen, which included an NSAID, an opioid medication, and an antiemetic. Possible NSAIDs included meloxicam or naproxen while possible opioids included combination hydrocodone-acetaminophen, combination oxycodone-acetaminophen, or oxycodone alone based on patient allergies, preferences, and tolerability. Regarding antiemetic agents, ondansetron was the typical medication prescribed. Patients were also encouraged to use over-the-counter acetaminophen in conjunction with the NSAIDs for postoperative pain control as a first-line treatment. In addition to these prescriptions, the senior author also included preoperative counseling regarding the dangers of opioid use, which included risks of addiction/substance abuse, respiratory depression, nausea, constipation, and mood disorders. Patients and their family members were counseled that there would be some discomfort after the surgery and narcotic medication should not be taken to be pain-free.

### Statistical Analysis

Baseline demographic characteristics were calculated and tabulated at the time of surgery. Means and SDs were calculated for the number of opioids prescribed, number of opioids reported taken, and number of days of opioid use. Percentages of reported leftover opioids and opioid retention were also calculated across the study cohort. The magnitude of opioid overprescribing was calculated by dividing the number of opioid pills prescribed by the number of opioid pills used. Bivariable linear regression was conducted to examine the relationship between opioid utilization and both opioids prescribed and number of NSAIDs taken. All statistical analyses were conducted using SPSS version 28 (IBM). Statistical significance was set at a *P*-value of <0.05. For regression analysis, a critical R value for the Pearson correlation coefficient was set at 0.155 for a *P*-value of 0.05 and 0.218 for a *P*-value of 0.01 given our sample size.^15^ Sample size for our regression was deemed adequate based on the rule of a minimum 10 participants per variable in the final regression model.^16^

## Results

During our study time frame, 157 patients were enrolled for survey administration. Of these individuals, 40 did not complete the survey after attempting to contact them and three individuals withdrew from the study, leaving a final included sample size of 114 (72.6%). The average age of our study population was 14.9 ± 2.2 years at the time of surgery, with 70 individuals (61.4%) being female and 44 (38.6%) being male. Most individuals underwent a unilateral procedure (91.2%). Knee procedures were most common in our study cohort in 78.1% of surgeries, with hip procedures comprising the remaining 21.9%. Most patients in our study underwent medial patellofemoral ligament (MPFL) reconstruction (24.6%), with cruciate/collateral ligament repair (21.9%) and hip arthroscopic procedures (21.9%) being the most notable other procedure types. Study cohort demographics are listed in Table 1.

**Table 1:** Demographics of the Study Cohort

Variable	Value^a^
Age, yr	14.9 (2.2)
Sex	
Female	70 (61.4%)
Male	44 (38.6%)
Laterality	
Unilateral	104 (91.2%)
Bilateral	10 (8.8%)
Surgery location	
Knee	89 (78.1%)
Hip	25 (21.9%)
Surgery type	
Cruciate/collateral ligament repair/reconstruction	25 (21.9%)
MPFL reconstruction	28 (24.6%)
Meniscus débridement/repair	13 (11.4%)
Tibial spine repair	4 (3.5%)
Hip arthroscopy	25 (21.9%)
Other^b^	19 (16.7%)

a Values reported as n (%), except for “age, years,” which is reported as mean (SD).

b Other included procedures such as osteochondritis dissecans of the knee drilling, osteochondral allograft placement, and hardware removal.

Metrics regarding opioid prescription and patient-reported opioid utilization retention are given in Table 2. Most patients were prescribed a combination of hydrocodone and acetaminophen (96.5%). Patients were prescribed a mean of 12.0 ± 5.0 opioid pills in the postoperative setting, additionally reporting utilization of a mean of 4.4 ± 6.1 pills over a mean of 2.7 ± 5.1 days. Calculating the magnitude of opioid overprescription by dividing the mean number of pills prescribed by the mean number of pills taken demonstrates that patients were prescribed 2.73 times the number of opioid pills used in the postoperative setting. In addition, 100 patients (87.7%) reported having unused opioid medication after their surgery. Of these individuals, 71 (71.0%) reported retention of these unused opioids in their home. Most patients did not require opioid refills (96.5%).

**Table 2:** Opioid Prescription and Patient-Reported Utilization

Variable	N (%) or Mean ± SD
Opioid prescribed	
Hydrocodone/acetaminophen 5/325 mg	100 (87.7%)
Hydrocodone/acetaminophen 5/300 mg	5 (4.4%)
Half tablets of hydrocodone/acetaminophen 5/325 mg	5 (4.4%)
Oxycodone/acetaminophen 5/325 mg	2 (1.8%)
Oxycodone 5 mg	2 (1.8%)
Total cohort	
Opioid pills prescribed	12.0 ± 5.0
Reported opioid pills taken	4.4 ± 6.1
Reported days of total opioid use	2.7 ± 5.1
No. of patients with refills	4 (3.5%)
Unused opioid medication	100 (87.7%)
Retention of unused opioids^a^	71 (71.0%)
Magnitude of opioid overprescription^b^	2.73

a Calculated as a percentage of individuals reporting unused opioid medication in the postoperative period.

b Calculated as the quotient of “opioid pills prescribed” and “reported opioid pills taken.”

Bivariable linear regression results are provided in Table 3. Notable associations were demonstrated regarding the number of opioid pills taken and both opioid pills prescribed and number of NSAID pills taken. Individuals reporting more opioid pills prescribed were associated with increased opioid consumption (β = 0.582, R = 0.471, *P* < 0.001). In addition, individuals reporting increased consumption of NSAIDs were associated with increased opioid consumption (β = 0.066, R = 0.266, *P* = 0.034).

**Table 3:** Bivariable Linear Regression of Opioid Usage

Variable	β (95% confidence interval)	R^a^	R^2b^	*P*
Opioid pills prescribed	0.582 (0.353, 0.812)	0.471	0.222	<0.001
NSAIDs taken	0.066 (0.005, 0.128)	0.266	0.071	0.034

a R = Pearson correlation coefficient.

b R^2^ = coefficient of determination.

## Discussion

Our results help further characterize opioid usage patterns in the postsurgical pediatric orthopaedic population, providing information regarding the number of opioid pills used versus the amount prescribed and a characterization of patient behavior regarding leftover opioid medications. Within our study cohort, there was relatively low utilization of opioids in the postoperative setting, with patients using less than five pills on average and most patients not seeking refills, leading to relative overprescribing of medications to these patients. Thus, we observed high rates of leftover opioid medications coupled with high community retention rates of these medications.

Overprescription of opioid medications in the orthopaedic surgery setting has been recently investigated and described. Capelle et al^7^ examined a cohort of 223 adult patients undergoing sports orthopaedic surgery and surveyed them for opioid usage versus their prescribed amount; similar to our study, they demonstrated that individuals were prescribed approximately 2.5 times the opioid pills needed for their postoperative analgesia. Tepolt et al^17^ investigated opioid utilization and overprescription in a cohort of 100 adolescents after knee arthroscopy and demonstrated that individuals in their study cohort were prescribed approximately three times the amount of opioid pills needed and used. Premkumar et al^18^ reported findings for a cohort of 103 patients who underwent total knee arthroplasty and were surveyed for opioid usage in the postoperative period, demonstrating that approximately two-thirds of their cohort had leftover opioids and only approximately one-fourth of those individuals reported properly disposing of the leftover medication. Bhashyam et al^19^ evaluated opioid use after foot and ankle procedures, demonstrating that patients in their cohort used a mean of 47.6% of prescribed opioid pills after surgery. A systematic review by Sheth et al^20^ found that overprescription of opioids was common after sports medicine procedures, with more than one-third of prescribed opioids remaining after utilization after surgery. Similar to the results of our study, Stone et al^21^ described opioid utilization patterns after pediatric ambulatory surgery and reported over 90% of individuals having leftover opioids, with only 42% reporting disposal of these medications. The aforementioned studies all provide similar results to this study, demonstrating that patients undergoing orthopaedic surgery vastly underutilize their prescribed opioids based on typical prescribing patterns, which opens up the potential for community retention and illicit diversion. Adjustments to prescribing patterns need to be implemented in this patient population, likely through limiting both the amount and recommended duration of opioid utilization, especially in the pediatric sports medicine population as investigated by our study.

A potential method to mitigate opioid retention is to implement either patient education or a convenient system for patients to dispose of these medications. Voepel-Lewis et al^22^ investigated the efficacy of providing a disposal method to parents after their child's surgery in combination with web-based education regarding opioid usage risks and the importance of disposal; this study demonstrated that the combination of these two elements was effective both at promoting prompt, appropriate disposal and reducing plans for opioid retention. Lawrence et al^23^ evaluated a strategy to promote excess opioid disposal by providing families of pediatric patients undergoing orthopaedic surgery with an opioid disposal bag of activated charcoal and instructions on opioid usage and disposal, finding that individuals in the intervention arm exhibited higher rates of opioid disposal than their counterparts. Similar to the study by Lawrence et al,^23^ Haverland et al^24^ demonstrated similar results when providing patients with an opioid disposal bag and patient education after gynecologic surgery. Implementation of similar disposal methods and patient education may have reduced the amount of opioid retention seen in our study population. Notably, patients in our study were not directly counseled regarding opioid disposal, which may provide an explanation for the high levels of opioid retention observed in our study population. Although our institution does offer opioid disposal sites, counseling on disposal was not a standard practice during the study period.

Another potential method to reduce opioid abuse is to reduce the number of opioids prescribed, which may be attainable through surgeon education. Increasing prescriber awareness of actual patient postoperative opioid utilization may help change physician prescribing patterns. Pursuing this surgeon-level education, in addition to appropriate patient-level education, may provide a multipoint approach to the reduction of opioid prescription, utilization, and retention.

## Limitations

There are several limitations of our study that warrant consideration. First, it should be noted that this study at hand assesses only patients from a single-surgeon practice at a large academic center, which may reduce the overall generalizability of the results of this study to other settings and patient populations. Biases inherent to survey studies that may influence our results are both the accurate recall of opioid utilization by our patients (recall bias) and the knowledge of patients that they would be receiving a survey regarding their opioid utilization (Hawthorne bias); these biases may have influenced patient responses because of the survey nature of this study. In addition, we were only able to collect surveys from 72.6% of our study population. Patients were also counseled preoperatively regarding the potential adverse effects of opioid utilization, which may have influenced utilization patterns in our study cohort.

## Conclusion

In pediatric patients undergoing sports medicine hip and knee surgery, the opioid medication utilization in the postoperative period averaged 4.4 ± 6.1 pills over 2.7 ± 5.1 days. Overall, our study results help characterize the utilization patterns of opioid medications in the postsurgical pediatric sports orthopaedic population and suggest that orthopaedic surgeons may be able to provide smaller quantities of opioid pills for analgesia than is typically prescribed, which in turn may help reduce the amount of prescription opioid medications present in the community.
